# Efficient use of sentinel sites: detection of invasive honeybee pests and diseases in the UK

**DOI:** 10.1098/rsif.2016.0908

**Published:** 2017-04-26

**Authors:** Matt J. Keeling, Samik Datta, Daniel N. Franklin, Ivor Flatman, Andy Wattam, Mike Brown, Giles E. Budge

**Affiliations:** 1 Zeeman Institute: SBIDER, University of Warwick, Coventry CV4 7AL, UK; 2 Mathematics Institute, University of Warwick, Coventry CV4 7AL, UK; 3 School of Life Sciences, University of Warwick, Coventry CV4 7AL, UK; 4 Animal and Plant Health Agency, Sand Hutton, York YO41 1LZ, UK; 5 Fera, Sand Hutton, York YO41 1LZ, UK; 6 Institute for Agri-Food Research and Innovation, Newcastle University, Newcastle upon Tyne NE1 7RU, UK

**Keywords:** invasion, eradication, pathogen, foulbrood, Tropilaelaps, small hive beetle

## Abstract

Sentinel sites, where problems can be identified early or investigated in detail, form an important part of planning for exotic disease outbreaks in humans, livestock and plants. Key questions are: how many sentinels are required, where should they be positioned and how effective are they at rapidly identifying new invasions? The sentinel apiary system for invasive honeybee pests and diseases illustrates the costs and benefits of such approaches. Here, we address these issues with two mathematical modelling approaches. The first approach is generic and uses probabilistic arguments to calculate the average number of affected sites when an outbreak is first detected, providing rapid and general insights that we have applied to a range of infectious diseases. The second approach uses a computationally intensive, stochastic, spatial model to simulate multiple outbreaks and to determine appropriate sentinel locations for UK apiaries. Both models quantify the anticipated increase in success of sentinel sites as their number increases and as non-sentinel sites become worse at detection; however, unexpectedly sentinels perform relatively better for faster growing outbreaks. Additionally, the spatial model allows us to quantify the substantial role that carefully positioned sentinels can play in the rapid detection of exotic invasions.

## 1. Introduction

The aim of surveillance in a biosecurity context is to monitor for changes to the health of human, animal or plant populations, and is essential to provide evidence of the absence of a disease or pest organism [1]. In addition, surveillance—either focused or applied en masse—is a key method of detecting novel or unexpected patterns, which may signify the invasion or emergence of new health issues [2]. Early interception is particularly important when considering the spread of an invasive pest or disease to a new territory, because timely detection may allow eradiation rather than establishment [3]. Sentinel surveillance concentrates activities on selected subpopulations to enhance detection and improve cost-effectiveness of surveillance efforts [4]. The term ‘sentinel’ invokes the concept of standing guard or keeping watch, and can be used in many different surveillance contexts. Sentinels can be deliberately placed, like the classic miner's canary [4], or they may be more observational, like crow deaths preceding human outbreaks of West Nile virus [5] or even simply selected locations with heightened levels of detection and effective reporting [6].

Here, we develop two distinct but complementary approaches to quantify the impact of intense-surveillance sentinel locations on the early detection of outbreaks. The first method is tractable and derives explicit mathematical formulae for the probability of detection given a general exponentially growing outbreak. The second method is tailored to honeybee pests and diseases in the UK, and allows us to assess the spatial aspect of outbreaks and hence the potential to choose optimal sentinel apiary locations. We assume that each apiary contains a constant number of hives throughout all time and all simulations, but there is considerable heterogeneity between apiaries as captured by National Bee Unit (NBU) recorded inspections. In both models, non-sentinel (normal) sites are assumed to detect an invasive species at a low constant rate; by contrast, at sentinel locations it is assumed that regular inspections are carried out which have a substantially higher chance of detecting an invading organism if it is present at the location. Therefore, in both models, the driving force is the rapidly increasing number of sites that are affected, increasing the likelihood both that one of the owners notices the pest/disease and that a sentinel is affected.

The global trade in honeybees and their hive products has resulted in the concomitant movement of their pests and parasites including: small hive beetle [7,8], Asian hornet [9], the microsporidium *Nosema ceranae* [10] and ectoparasitic mites *Varroa destructor* [11] and *Tropilaelaps* spp. [12]. Each invasion can affect the health of the resident honeybee population, leading to large-scale colony deaths (e.g. *Varroa* [11] and *Nosema* [13]). Failed early eradication can result in long-term impacts on pollinator health and necessitate continued efforts on containment and mitigation, both of which have significant economic consequences [3,14]; for example, the cost of failing to eradicate *Varroa* in the UK has been estimated at over £27 million annually [15]. To enable the early detection and eradication of exotic honeybee parasites the NBU already supports a network of 131 sentinel apiaries across England and Wales.

Our aims in this paper are to explore the generalities of using sentinel locations to rapidly detect invading organisms, considering under which circumstances sentinels are most likely to offer a substantial benefit. These concepts are then extended to the specific problem of honeybee pests and pathogens, and we predict the impact of sentinel apiaries and how their spatial arrangement could be improved.

## 2. The tractable model

Both the mathematically tractable model and the spatial simulation adopt the same assumptions about detection and the action of sentinels, based on the probability of initially detecting a novel invasion (at sentinel and non-sentinel locations). For non-sentinel locations, we assume that there is a daily independent probability, *p*, that infection is identified at each (infected) location and reported to the authorities. Sentinel locations are assumed to be at a proportion, *s*, of potential sites and are periodically inspected, with a time *T* between inspections. Throughout this work, we assume that the periodic inspection of a sentinel location always correctly identifies infection, and that infection is immediately detectable; both of these assumptions help to clarify the results and reduce the number of model parameters, but have limited qualitative impact on the efficacy of sentinels (see the electronic supplementary material).

**Figure 1.**
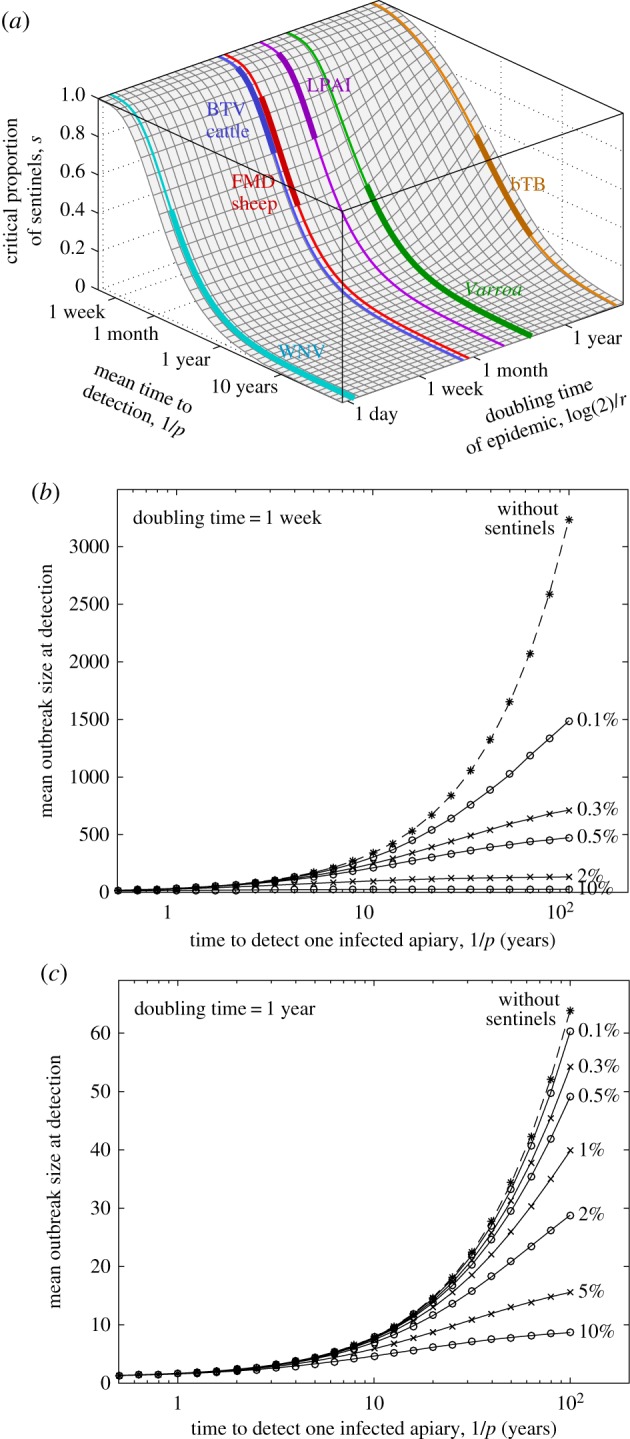
Theoretical prediction of outbreak sizes at detection. (*a*) The critical proportion of sentinels required to be equal to owner detection; coloured lines show the estimated doubling times for five diseases: blue tongue virus (BTV) in cattle [16], West Nile virus (WNV) in wild birds [17], foot-and-mouth disease (FMD) in sheep [18], low-pathogenicity avian influenza (LPAI) in chickens [19], *Varroa* in honeybees and bovine tuberculosis (bTB) in GB cattle herds [20]. Lines correspond to the doubling time given by epidemiological parameters from the literature, while thick lines correspond to approximate detection rates by owners. (*b,c*) Impact of low proportions of sentinels on the size of an outbreak at the time of detection, measured as the number of infested apiaries. For faster growing epidemics (*b*) the outbreaks are larger but sentinels have greater impact. (Throughout we assume the time between surveillance visits for sentinels *T* = 28 days.)

From these assumptions, relatively simple algebraic manipulation generates an explicit formula for the probability of first detection of an epidemic on a given day assuming exponential growth of new cases (see the electronic supplementary material). In general, this probability is a function of four variables (*p*, *s*, *T* and the growth rate of the epidemic, *r*) as well as time. However, examining the parameter conditions when the sentinels are equally likely to detect infection as non-sentinels, we can parsimoniously quantify the proportion of locations that need to be sentinels to have a substantive impact on the early detection of a novel outbreak (figure 1*a*). It is clear, and intuitive, that sentinel locations are most effective at detecting outbreaks where individual infections are likely to remain undetected for long periods of time (*p* small), when even a low number of sentinel locations can be highly beneficial. Additionally, outbreaks that grow more slowly (*r* small) require higher proportions of sentinels for them to be equal to random detection.

Picking a specific epidemic growth rate (*r*), we can more readily quantify the potential impact of a small proportion of sentinel locations. In particular, we compare outbreaks that double over different time periods (one week in figure 1*b* or 1 year in figure 1*c*). Mean time to detection at a non-sentinel location (1/*p*) is again the key determinant of the expected outbreak size (at the point of detection), with the growth rate of the outbreak (*r*) playing a secondary but significant role. Of more applied interest is the impact of a small proportion of sentinels (*s* < 10%). For slow growing outbreaks (figure 1*c*) a few sentinels (*s* < 0.5%) make a limited difference; however, for more rapid outbreaks (figure 1*b*) even having just a thousandth of the population as sentinels (*s* = 0.1%) substantially reduces the size of the outbreak, especially when non-sentinel detection times are long.

Such simple mathematical models therefore suggest that sentinel locations can have a substantial practical benefit, although arguably a concerted effort to improve education and awareness such that non-sentinel infections are detected faster (1/*p* is decreased) may be a more efficient use of resources, despite the practical difficulties associated with achieving such a policy.

## 3. The spatial model

**Figure 2.**
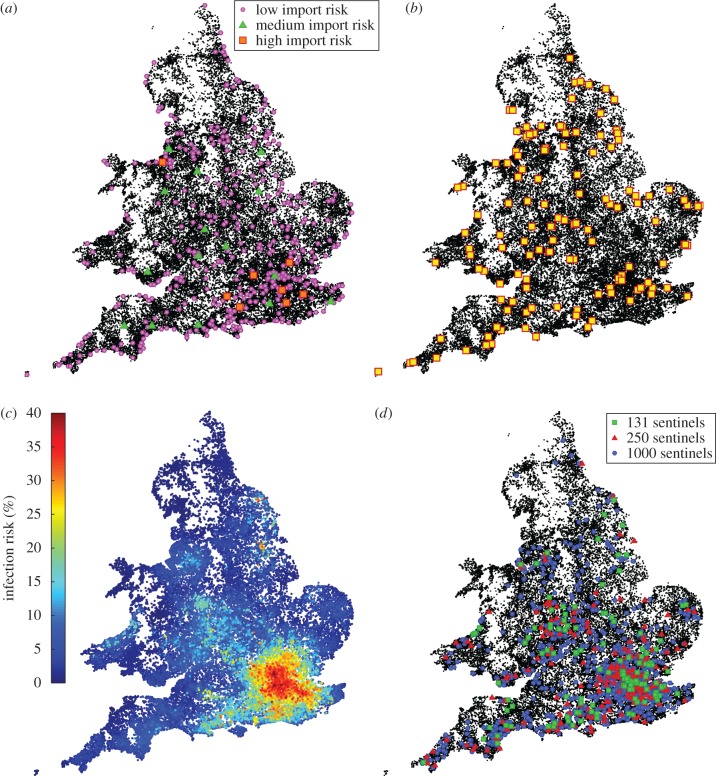
Inputs and results of the stochastic spatial model. (*a*) Locations of apiaries (black dots) in England and Wales together with potential import locations catagorized by risk (low risk, pink; medium risk, green; high risk, red; see Methods). (*b*) Position of the 131 sentinel apiaries in England and Wales. (*c*) Predicted average risk of infection for each apiary after 3 years of uncontrolled growth where the seed of infection is picked randomly from import risk locations. (*d*) Improved location of sentinels, for different numbers of sentinel apiaries (131, green; 250, red; 1000, blue).

The mathematical model defined above provides considerable generic understanding of the impact of sentinel locations, but lacks multiple features of the real-world problem. Most notably, many epidemics have a strong spatio-temporal component [21], with infection often spreading as a stochastic wave [22]. This is in direct contrast to the analytical model above which assumed a simple exponential increase and randomized infection of locations. In addition, sentinels are generally not randomly selected from the entire population, but are strategically chosen to maximize the chance of detecting an infection early. Here, we use the detection of pathogen invasion into the honeybee population of England and Wales as a well-defined data-rich spatial problem that has wide-ranging implications, although the decline in honeybees, often as a result of invasive pests, is a worldwide problem. We now consider the results of stochastic spatial simulations (figure 2) parametrized to match recorded patterns of invasion and disease (the electronic supplementary material offers a full description of the model). Our aim was to select sentinel apiaries that minimize the average outbreak size. This is a computationally demanding process, which in general would require many simulations for each potential sentinel apiary configuration to evaluate the expected impact, to be repeated for multiple configurations. However, given that we are only interested in the behaviour up to the point of detection, and that no specific controls can be applied before detection, this computational burden can be greatly reduced. By storing the data from multiple stochastic epidemics (in the absence of controls, sentinels and owner detection) the chance of detection at any given time point can be calculated numerically for any configuration of sentinel apiaries or parameters for owner detection.

The simulations use the known location of apiaries in England and Wales and capture local transmission, infection dynamics within an apiary and long-range transmission between apiaries owned by the same beekeeper [23]. The parameters are chosen to capture the observed spatial spread of multiple honeybee diseases, and lead to epidemics that spread over a period of years, with an early doubling time of around four to six months. All simulations are begun with 10 infected apiaries chosen to be in close proximity (within 10 km) to locations that are considered to represent a potential risk of importing a novel pest or disease (figure 2*a*). In decreasing order of risk, these locations are: package bees/nucleus importers, queen importers, imported honey packers, hive products importers, fruit and vegetable wholesale markets, zoos, plant importers, freight depots or ports, airports, quarantine facilities (all shown as coloured dots in figure 2*a*), apiaries along the south coast, and all apiaries (see the electronic supplementary material for a greater description of these import locations). The current selection of 131 sentinel apiary locations provides a relatively uniform coverage of England and Wales, which equalized the demands on regional bee inspectors (figure 2*b*).

The risk of any apiary being infected at the end of a 3 year simulation (figure 2*c*) closely mirrors the distribution of import risks. Hence the hotspots for infection closely match the large urban areas in the Midlands, the northeast of England and Manchester, with London dominating. We stress that this pattern is predominantly driven by the risk of imports into the regions, rather than preferential spread to these regions as the simulated epidemics progress. Given these results, it is unsurprising that the most effective distributions of sentinels also mimics this pattern, with many more sentinels in the southeast of the country (figure 2*d*). With over 50 000 apiaries in England and Wales an exhaustive search of all possible sentinel apiary configurations is impossible and therefore we cannot state that the locations predicted are truly optimal; however, they represent a substantial improvement over random placements—although individual positions should be finessed by local knowledge.

**Figure 3.**
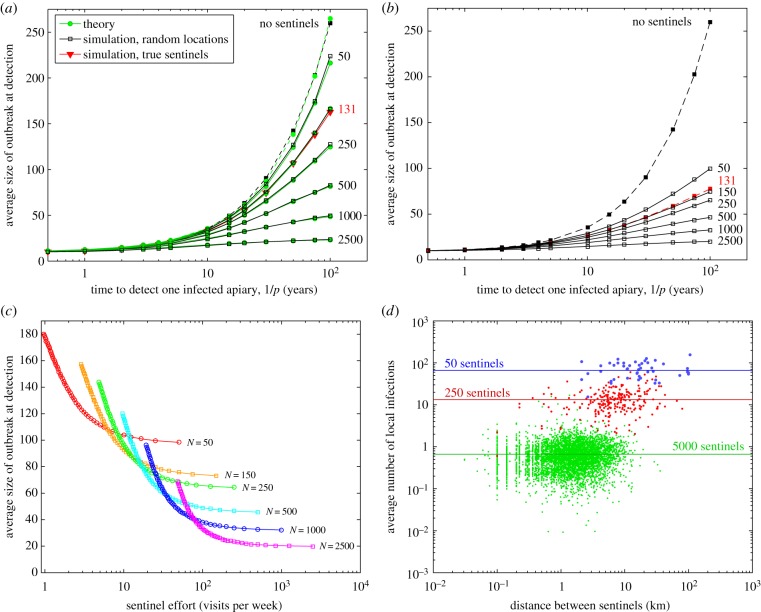
Comparison of theory and simulation. (*a*) Predicted impact of randomly located sentinel apiaries in the stochastic simulation model (black lines) and from the theoretical model (green line), together with the simulated impact of the current pattern of 131 sentinel apiaries (red). (*b*) Impact of improved sentinel locations, highlighting the potential benefits of relocating the current 131 sentinel apiaries (red). (*c*) The effects of inspection frequency and number of sentinels for a fixed effort, when owner detection is very slow (1/*p* = 100 years). (*d*) The optimal location of sentinels is largely determined by a need to equalize the expected number of infections that are nearest to each sentinel. (Throughout we assume the time between surveillance visits for sentinels *T* = 28 days.)

Figure 3*a* compares the results of the theoretical model (green dots, see figure 1) with a random placement of sentinels in the full spatial model (black squares); despite the extreme differences in modelling approach, both methods are in close agreement. However, more striking is that the current placement of sentinels (red triangles) has an impact that is indistinguishable from a random placement of the same number of sentinel apiaries. By contrast, using a carefully chosen set of sentinel apiaries more closely linked to import risk locations can substantially reduce both the time to detection and the expected size of the outbreak at the moment of detection (figure 3*b*). In fact, careful spatial placement of 131 sentinels performs as well as random placement of around 500 sentinels, generating a huge improvement in efficiency. This is to be expected—not only are the locations of sentinels chosen to substantially increase the risk of detection, but also the choice of initial seeding based on risk locations greatly constrains the patterns of greatest infection.

Throughout we have assumed that sentinel inspections take place regularly, with inspections scheduled on a monthly basis—which matches brood time scales within the hive. However, a key question concerns the optimal use of sentinels in a resource-limited setting: is it better to repeatedly visit highest-risk sentinel apiaries or visit a larger number less frequently? Assuming very slow owner detection rates (1/*p* = 100 years, such that without sentinels it takes 2–3 years to detect an infection), the impact of different combinations of sentinel numbers and inspection interval is assessed (figure 3*c*). For a given amount of effort (inspections per week), an intermediate trade-off between numbers and frequency is optimal; visiting the chosen sentinels three to five times in a season in general gives the greatest impact.

Finally, we return to the question of whether there are any general insights governing the optimal arrangement of sentinels. We tessellate the landscape by assigning each apiary to the nearest sentinel location, and examine the properties of all apiaries local to each sentinel. We find that the expected amount of infection local to each sentinel remains relatively constant even as the spacing between sentinels changes (figure 3*d*). We note that sentinels associated with relatively low levels of infection in their local environment are generally within clusters of high risk promoting detection in these areas. This suggests, as may be expected, that the sentinels are arranged to inform about similar levels of infection in their surrounding environment; this may provide a simpler mechanism for determining the placement of sentinels in general.

## 4. Discussion

Invasive pests and diseases are a major threat to the health of human, livestock and wildlife populations. Key to the control of such novel invasions is early detection, allowing controls to be enacted while the pathogen is still at low numbers [24,25]. Sentinel surveillance may have a crucial role to play in this early detection [26]. As shown here, fast growing invasions which are difficult to detect in the general population substantially benefit from the presence of sentinels; additionally, an understanding of risk factors can provide substantial benefits from a refined spatial arrangement of sentinel locations.

There are considerable risks to UK honeybees posed by invading pests, pathogens and parasites of bees, including: *Tropilaelaps* mites, small hive beetle (which invaded Italy in 2014) and Asian hornet (which arrived in the UK in 2016). Early detection of such invasions is a key element of control, and our models suggest that this can be achieved in two different ways. Greater vigilance on the part of the general population (reducing the mean time to detection 1/*p* in the non-sentinel population) has a dramatic impact on detection. This suggests that education and awareness campaigns may be a key tool in the fight against invaders. However, such national campaigns may be costly and a full cost–benefit analysis would need to be conducted to compare the cost-effectiveness.

We demonstrate that careful positioning of sentinels across the landscape (reflecting the most probably introduction points [27]) can have a profound influence on early detection, greatly improving on random or uniformly distributed inspection locations—a facet we postulate will hold for any host species and invasive pests or pathogens. This rapid detection is contingent on good geographical knowledge of where pests are likely to invade. We find that sentinel apiaries should largely reflect the spatial pattern of import risks, which clusters in the southeast of England and around London. In particular, if we associate each apiary with its nearest sentinel, then the optimal pattern has approximately equal levels of infection associated with its neighbouring apiaries—providing a generic means of positioning with a minimum of computational effort.

These analyses raise the critical applied question of how many sentinel apiaries the UK needs. This is a value judgement, balancing the cost of sentinel apiaries against the risk of a novel outbreak remaining undetected. It is clear from figures 1 and 3 that even a limited number of sentinel apiaries can be highly beneficial for preventing large-scale undetected outbreaks. It is also apparent that having many thousands of sentinel apiaries is both impractical and unlikely to generate huge additional improvements. This suggests advantages to a carefully parametrized cost–benefit analysis to balance continual costs of operating a network of sentinel apiaries against the stochastic benefits of early detection and therefore more likely and less expensive eradication of invasive pests and pathogens.

## Supplementary Material

Supplementary Material

## References

[1] Salman MD 2003 Surveillance and monitoring systems for animal health programs and disease surveys. Ames, IA: Iowa State Press.

[2] Fricker RD 2013 Introduction to statistical methods for biosurveillance. Cambridge, UK: Cambridge University Press.

[3] Heersink DK, Caley P, Paini DR, Barry SC 2015 Quantifying the establishment likelihood of invasive alien species introductions through ports with application to honeybees in Australia. Risk Anal. 36, 892–903. (10.1111/risa.12476) 26482012

[4] Burrell GA, Seibert FM 1916 Miners' Circular 14, 1–23. See https://hdl.handle.net/2027/mdp.39015077561960.

[5] Eidson M, Komar N, Sorhage F, Nelson R, Talbot T, Mostashari F, McLean R, the West Nile Virus Avian Mortality Surveillance Group. 2001 Crow deaths as a sentinel surveillance system for West Nile virus in the Northeastern United States, 1999. Emerg. Infect. Dis. 7, 615–620. (10.3201/eid0704.017402) 11585521 PMC2631775

[6] Bolotin S, Pebody R, White PJ, McMenamin J, Perera L, Nguyen-Van-Tam JS, Barlow T, Watson JM 2012 A new sentinel surveillance system for severe influenza in England shows a shift in age distribution of hospitalised cases in the post-pandemic. PLoS ONE 7, e30279 (10.1371/journal.pone.0030279) 22291929 PMC3264602

[7] Neumann P, Pettis JS, Schäfer MO 2016 Quo vadis *Aethina tumida*? Biology and control of small hive beetles. Apidologie 47, 427–466. (10.1007/s13592-016-0426-x)

[8] Cuthbertson AG, Wakefield ME, Powell ME, Marris G, Anderson H, Budge GE, Mathers JJ, Blackburn LF, Brown MA 2013 The small hive beetle *Aethina tumida*: a review of its biology and control measures. Curr. Zool. 59, 644–653. (10.1093/czoolo/59.5.644)

[9] Villemant C, Haxaire J, Streito J-C 2006 First assessment of *Vespa velutina* Lepeletier spread in France (Hymenoptera, Vespidae). Bull. Soc. Entomol. France 111, 535–538.

[10] Higes M, Martín R, Meana A 2006 *Nosema ceranae*, a new microsporidian parasite in honeybees in Europe. J. Invertebr. Pathol. 92, 93–95. (10.1016/j.jip.2006.02.005) 16574143

[11] Rosenkranz P, Aumeier P, Ziegelmann B 2010 Biology and control of *Varroa destructor*. J. Invertebr. Pathol. 103, S96–S119. (10.1016/j.jip.2009.07.016) 19909970

[12] Anderson DL, Morgan MJ 2007 Genetic and morphological variation of bee-parasitic Tropilaelaps mites (Acari : Laelapidae): new and re-defined species. Exp. Appl. Acarol. 43, 1–24. (10.1007/s10493-007-9103-0) 17828576

[13] Higes M et al. 2008 How natural infection by *Nosema ceranae* causes honeybee colony collapse. Environ. Microbiol. 10, 2659–2669. (10.1111/j.1462-2920.2008.01687.x) 18647336

[14] Clifford D, Barry S, Cook D, Duthie R, Anderson D 2011 Using simulation to evaluate time to detect incursions in honeybee biosecurity in Australia. Risk Anal. 31, 1961–1968. (10.1111/j.1539-6924.2011.01607.x) 21449958

[15] Williams F et al. 2010 The economic cost of invasive non-native species on Great Britain. CABI Project no. VM10066. Wallingford, UK: CABI.

[16] Sanders CJ, Shortall CR, Gubbins S, Burgin L, Gloster J, Harrington R, Reynolds DR, Mellor PS, Carpenter S 2011 Influence of season and meteorological parameters on flight activity of *Culicoides* biting midges. J. Appl. Ecol. 48, 1355–1364. (10.1111/j.1365-2664.2011.02051.x)

[17] Wonham MJ, de-Camino-Beck T, Lewis MA 2004 An epidemiological model for West Nile virus: invasion analysis and control applications. Proc. R. Soc. Lond. B 271, 501–507. (10.1098/rspb.2003.2608) PMC1691622 15129960

[18] Tildesley MJ, Deardon R, Savill NJ, Bessell PR, Brooks SP, Woolhouse MEJ, Grenfell BT, Keeling MJ 2008 Accuracy of models for the 2001 foot-and-mouth epidemic. Proc. R. Soc. B 275, 1459–1468. (10.1098/rspb.2008.0006) PMC2376304 18364313

[19] Akey BL 2003 Low-pathogenicity H7N2 avian influenza outbreak in Virginia during 2002. Avian Dis. 47, 1099–1103. (10.1637/0005-2086-47.s3.1099) 14575120

[20] Brooks-Pollock E, Roberts GO, Keeling MJ 2014 A dynamic model of bovine tuberculosis spread and control in Great Britain. Nature 511, 228–231. (10.1038/nature13529) 25008532

[21] Mollison D 1977 Spatial contact models for ecological and epidemic spread. J. R. Stat. Soc. B 39, 283–326.

[22] Grenfell BT, Bjornstad ON, Kappey J 2001 Travelling waves and spatial hierarchies in measles epidemics. Nature 414, 716–723. (10.1038/414716a) 11742391

[23] Datta S, Bull JC, Budge GE, Keeling MJ 2013 Modelling the spread of American foulbrood in honeybees. J. R. Soc. Interface 10, 20130650 (10.1098/rsif.2013.0650) 24026473 PMC3785836

[24] Fraser C, Riley S, Anderson RM, Ferguson NM 2004 Factors that make an infectious disease outbreak controllable. Proc. Natl Acad. Sci. USA 101, 6146–6151. (10.1073/pnas.0307506101) 15071187 PMC395937

[25] Veitch CR, Clout MN 2002 Turning the tide: the eradication of invasive species. IUCN SSC Invasive Species Specialist Group. Cambridge, UK: IUCN.

[26] Arita I, Nakane M, Kojima K, Yoshihara N, Nakano T, El-Gohary A 2004 Role of a sentinel surveillance system in the context of global surveillance of infectious diseases. Lancet Infect. Dis. 4, 171–177. (10.1016/S1473-3099(04)00942-9) 14998504 PMC7129469

[27] Cannon R 2009 Inspecting and monitoring on a restricted budget—where best to look? Prev. Vet. Med. 92, 163–174. (10.1016/j.prevetmed.2009.06.009) 19640598

