# Supplementary material for: Efficient use of sentinel sites: detection of invasive honeybee pests and diseases in the UK
Source: J R Soc Interface. 2017 Apr 26;14(129):20160908. doi: 10.1098/rsif.2016.0908 (PMC5414905; doi:10.1098/rsif.2016.0908)
Supplement: Supplementary Material [file rsif20160908supp1.pdf]

Supplementary Materials for

Efficient Use of Sentinel Sites: detection of invasive honey bee pests and diseases in the UK.

Matt J. Keeling[1,2,3★], Samik Datta[1,2], Daniel N. Franklin[1,3], Ivor Flatman[4], Andy Wattam[4], Mike Brown[4] and Giles E. Budge[5,6★]

[1] Zeeman Institute: SBIDER, University of Warwick, Coventry, CV4 7AL.
[2] Mathematics Institute, University of Warwick, Coventry, CV4 7AL.
[3] School of Life Sciences, University of Warwick, Coventry, CV4 7AL.
[4] Animal and Plant Health Agency, Sand Hutton, York, YO41 1LZ.
[5] Fera, Sand Hutton, York, YO41 1LZ.
[6] Institute for Agri-Food Research and Innovation, Newcastle University, Newcastle upon Tyne, Tyne and Wear NE1 7RU.

⋆. Corresponding authors: m.j.keeling@warwick.ac.uk; giles.budge@fera.co.uk

1. Underlying Mathematics for Exponentially Growing Infections:

The fundamentals of both models can be best expressed in terms of the probability of detecting a novel invasion (at sentinel and non-sentinel locations) on a given day, assuming it has not already been detected. Throughout we assume that the probability of first detecting an invader is an independent random process.

Although we acknowledge that many of the mechanisms operate on a daily cycle, to simplify the mathematics we use a continuous time formulation. In particular, we assume that at time $t = 0$ an invasion occurs infecting I_0 locations; from this point the number of infected locations grows exponentially:

$$Z(t) = \begin{cases} I_0 \exp(rt) & t \geq 0 \\ 0 & t < 0. \end{cases}$$

The rate at which new infections are created is therefore

$$I(t) = \frac{dZ}{dt} = \begin{cases} I_0 r \exp(rt) + I_0 \delta(t) & t \geq 0 \\ 0 & t < 0, \end{cases}$$

where δ is the Dirac delta function, which accounts for the sudden invasion at time $t = 0$.

The probabilities for detection by owners, and because of sentinel surveillance, before a given time can then be written as

$$P_{\text{non}}(\text{detected before } t) = 1 - (1-p)^{\int_0^t Z(s)ds}$$

$$P_{\text{sentinel}}(\text{detected before } t) = 1 - (1-s)^{Q(t)}$$

$$\text{where } Q(t) = \begin{cases} \int_0^{t-T} I(u)du + \int_0^T \frac{u}{T} I(t-u)du & \text{when } t > T \\ \int_0^t \frac{u}{T} I(t-u)du & \text{otherwise,} \end{cases}$$

where *p* is the daily probability of detection by an owner, *s* is the proportion of the locations that are sentinels and *T* is the time between subsequent inspections of sentinel apiaries. Generally we assume that T=28 days in the main paper, although figure 3C gives the sensitivity to this assumption. *Q*(*t*) is all those infected locations that would have been inspected since they were infected if they were sentinels; hence the first term in Q (assuming *t*>*T*) is all those infected locations that occurred a sufficiently long time ago that they are guaranteed to have been inspected, while the second term measures the number of more recent infections that should have been inspected.

Setting these two probabilities equal and taking logs gives:

$$\frac{I_0}{r}\log(1-p)[\exp(rt)-1] = I_0 \log(1-s)\exp(rt)\frac{1}{rT}[1-\exp(-rT)]$$

$$\log(1-s) = \frac{\log(1-p)[1-\exp(-rt)]T}{[1-\exp(-rT)]} \qquad (1)$$

$$s \approx 1-(1-p)^{\frac{T}{[1-\exp(-rT)]}}. \qquad (2)$$

Equation (1) therefore defines a relationship between the proportion of locations that need to be sentinels (*s*) and other parameters, which lead to sentinel detection being as effective as non-sentinel detection. Figure 1 of the main text shows the shape of equation (1) as *r* and *p* are varied.

Alternatively looking at the mean number of cases at the point of detection gives

$$\text{Mean} = I_0 \frac{A[\ln(A)-1]}{\ln(A)}(1-p)^{-\frac{1}{r}} \quad \text{where } A \approx [(1-p)(1-s)]^{\frac{I_0}{r}}.$$

This is an exact expression (as long as the expected detection time at a non-sentinel site is longer than the surveillance period, *T*). If we can take the limit as *T* becomes relatively short, and make the further assumption that *p* and *s* are small we obtain

$$\text{Mean} = \frac{r}{p+s}\left(1+\frac{p}{r}\right)\left(1-\left[\frac{I_0(p+s)}{r}\right]^2\right),$$

indicating that the initial number of infected locations (I_0) has a limited impact, while to first order *s* and *p* are balanced.

1.1. Extensions to the timing within the tractable model.

A number of factors could be included in the above formulation to improve its accuracy. Many of these involve a greater realism in terms of time to detection and probability of detection, as detailed below:

- If there exists a fixed incubation period (*P*) between infection and the pest or pathogen being detectable, then the quantities $Z(t)$ and $I(t)$ can be redefined as the number of detectable infected locations, and the probability of detection equation remains unaffected, although the number of infected locations at the point of detection will require an additional factor of $\exp(rP)$.

- The above calculations assume that inspectors always identify infected apiaries when they are inspected. If inspectors only identify infected apiaries with probability q, and if this is an independent probability on each inspection, then the sentinel equation can be re-written as

$$P_{\text{sentinel}}(\text{detected before } t) \approx 1 - (1 - q\, s)^{Q(t)},$$

  assuming that s is small. As such, imperfect inspections are equivalent to a reduction in the proportion of sentinel sites. While these false negatives are easily accounted for, false positives are more complex and problem specific, in general it is to be hoped that further investigation of newly detected sites would uncover such errors. Such false positives are likely to inflate the cost and reduce confidence in the system.

- Finally, if we are dealing with an infestation that grows slowly within an apiary, this could be incorporated by insisting that the probably of detection (by both owners and inspectors) is reduced by a multiplicative factor $M(\tau)<1$, for time τ since infection. Here we expect M to start small, but grow to one as time advances and the level of infestation in the apiary increases. Assuming that p and s are small, this can be incorporated by replacing the number of infected apiaries $Z(t)$ with a time-lagged version $Y(t)$:

$$Y(t) = \int_0^t M(t-s) I(s) ds$$

  Therefore, while the effects of such time-varying detection rates are not immediately easy to quantify, we expect very similar results to the introduction of a fixed incubation period.

2. The Simulation Model.

The simulation model uses 54023 apiaries in England and Wales as the landscape across which the pest or pathogen can spread. We capture spread via both spatial proximity and ownership – linking apiaries with the same beekeeper. A spatially homogeneous landscape is assumed for the former, and parameters for both routes of transmission based on those parameters inferred from the invasion of *Varroa*. In particular, *SI* (susceptible-infectious) dynamics are assumed, with the risk of infecting a particular apiary, *a*, given by

$$Risk_a(t) = (h_a)^p \sum_{b \in \text{Infected}(t)} (h_b)^q K(a,b) \tau(t - \tau_b)$$

where *h* is the number of hives in each apiary, *p* and *q* control the non-linear nature of susceptibility and transmission, *K* is the distance based transmission kernel and τ accounts for increasing transmission with time since the date of infection (s). In particular,

$$\tau(s) = \frac{1}{1 + \exp(A - Bt)}, \qquad K(a,b) = \beta_D \frac{D^2}{D^2 + d_{a,b}^2} + \beta_N O_{a,b}$$

where $d_{a,b}$ is the distance between two apiaries and $O_{a,b}$ is one if the two apiaries have the same owner. Parameters are given in the table below.

Supplementary Table 1: Parameters used in the spatial infection model.

Parameter	Meaning	Value
A	Determines within apiary infection dynamics	4
B	Determines within apiary infection dynamics	0.8 per week
D	Spatial scale within transmission kernel	3.98km
β_D	Transmission rate due to distance	1.5×10^{-4} per wk
β_N	Transmission rate due to ownership network	2.1×10^{-4} per wk
p	Non-linear susceptibility, with number of hives in apiary	0.7
q	Non-linear transmission, with number of hives in apiary	0.3

We note that this is a relatively simple model of the dynamics of invasion within UK apiaries; for many invading pests and pathogens there will be complex within-hive dynamics which will influence both the probability of transmission and the probability of detection (by either owners or inspectors). However, our aim here is not to capture in the detail the behaviour of a particular invader, but to have a generic framework that captures the predominate elements of invasion and local disease spread. The above equations and parameters from the table reproduce dynamics that are broadly comparable (in terms of numbers of infested apiaries and spatial scale) with the Varroa invasion of the UK.

The infection is initiated in the vicinity of a particular location, which is chosen from three distinct groups:

1. One of the exotic risk points listed by the National Bee Unit;
2. Along the south coast of England, to simulate a pest or pathogen arriving either by sea or travelling over from the near continent;
3. Randomly at any apiary in the system.

Ten apiaries within ten km of the chosen location are infected at the start the epidemic. It was decided to infect ten apiaries to minimise the chance of stochastic extinction and to

minimise the stochastic variability between simulations, while still allowing the invasion to grow exponentially. Examining the tractable model, we find that the initial condition has a relatively small impact especially when outbreak sizes are expected to be large. Although it would have been possible to have invasion conditions that more accurately reflect the likely behaviour of a given pest or pathogen, we consider our assumption of a low constant number to be the most general and parsimonious. Following invasion the disease is allowed to spread unchecked for 5 years, and the infection status of all apiaries is recorded over time. In total, 130,000 simulations were recorded, for use in optimising the location of sentinels.

2.1. Optimising the Location of Sentinels.

The dynamics of multiple replications of the simulation model are stored to provide lists of when each apiary is infected. Given the infection times t_a for apiary *a* (assumed to be infinite if the apiary remains uninfected); the expected time of detection and the expected number of infected apiaries at that time can be explicitly calculated:

$$P_{\text{non}}^{\text{sim}}(\text{detected before } t) = 1 - (1-p)^{\sum_{\tau \le t} \#(t_a \le \tau)}$$

where $\#(t_a \le s)$ refers to the number of apiaries detected before time *s*.

$$P_{\text{sentinel}}^{\text{sim}}(\text{detected before } t) = \begin{cases} 1 & t_a < t - T \text{ for any } a \in \text{Sentinels} \\ 1 - \prod_{t_a < t} \dfrac{T + t_a - t}{T} & \text{otherwise} \end{cases}$$

The expected number of infected apiaries at the point of detection is then computed from the probabilities and the known number of infections:

$$E^{\text{sim}}(\text{infections at detection}) = \sum_t [P^{\text{sim}}(\text{detected before } t) - P^{\text{sim}}(\text{detected before } t-1)]\#(t_a \le t)$$

For a known set of infection times, this expected quantity (averaged over multiple simulations) can be calculated with great computational efficiency.

Optimisation of the sentinel locations is then performed using a genetic algorithm. Sentinels are initially randomly assigned to existing apiary locations, weighted in favour of apiaries that (on average) are infected earlier in simulations. Sets of sentinels are then selected based on their expected benefit (reduction in infected apiaries at the time of detection), the choice of sentinel locations from sets combined, and new locations trialled. Given the huge number of combinations, the true optimal set of sentinels is unlikely to be discovered, but the expected benefit rapidly saturates demonstrating a considerable advantage of the chosen set of sentinels over a random choice.

3. Spatial Risk of Infection

A scoring system was developed to reflect the relative risk of any exotic pests or disease occurring at random in the apiary network (score of 1) versus the risk at specific locations (up to 100 times). Evidence was derived from peer-reviewed literature, comprehensive Pest Risk Assessments (PRAs) and expert opinion from senior bee health inspectors. Quantification of these risk factors are given in Supplementary Table 2.

Foulbrood: Previous exotic outbreaks have been attributed to honey bee importers where brood might be present[1] or honey bee packers and the respective landfill sites where barrels are disposed[2]. Expert opinion also suggested a slight increase in risk when importing hive products such as pollen, wax or propolis, and that risks would pervade over 5 km as a conservative mean foraging distance of a honey bee in the UK[3].

***Tropilaelaps* spp.**: These ectoparasitic mites are not free-living and are almost exclusively confined to the brood of infested colonies, only spreading naturally during brief transport on adult bees[4]. Risk can be associated with honey bee importers where brood might be present, however the risk is low because importation is illegal from territories where the mite is known to be present. Therefore we assume the small size of these mites could lead to unreported movements to new areas. Local movement was assumed to be restricted to 5 km on the back of foraging worker bees.

Small hive beetle: In the USA it is not clear whether single or multiple introductions occurred[5,6], but the rapid spread is likely to be as a result movement of infested colonies, queen bees, packaged bees and beekeeping equipment, and also migratory beekeeping[7]. The first confirmed case of small hive beetle in Canada (Manitoba) in 2002 was at a wax rendering plant – the beetles being brought into country in beeswax cappings (unprocessed beeswax) from Texas, USA[8]. Crude hive product importers therefore scored highly for risk. Our assumption is that movement of the pest is also possible in trade of fruit, soil and compost with plants; anything that the beetle can survive in as adults, larvae, pupae or eggs. Sites having such products were also rated high risk (fruit and wholesale markets; zoos which use imported fruit, and plant importers due to concomitant soil movements). SHB may have arrived in the Southern States in the US by fruit importation into Charleston port, therefore freight ports and deports were seen as high risk. Honey bee imports were seen as high risk after SHB larvae were identified in a consignment of queen bees imported into Portugal from Texas[9]. It addition, because beetles have been seen travelling in a swarm, those importing package bees and nucleus colonies were seen as the highest possible risk. Military airports were seen as higher risk than civilian airports because of their ability to move soil on tracked vehicles. US military airports were seen as higher risk than UK military airports because of the propensity to move supplies and equipment from the US, which is infested with the SHB. Adult small hive beetles are strong fliers and are capable of flying over 10 km[10] and so a distance of 15 km was assumed (Supplementary Table 2). A comprehensive pest risk assessment is available for SHB which summarises much of this information[11].

The government funded National Bee Unit operate an existing Sentinel Apiary Network comprising 131 apiaries. Sites were chosen in part at random and in part proximal to locations of increased exotic risk[12,13]. The apiary owners receive training to help them identify exotic pests and diseases, conduct detailed surveys biannually, send in samples for

diagnostic testing and are provided with equipment to trap and/or help identify non-native pests. These measures are designed to increase the likelihood of reporting new exotic incursions[12]. In addition, running in parallel to the sentinel apiary network, the National Bee Unit conducts exotic surveillance inspections at random and apiaries deemed to have increased exotic risk[12], this represents around 25% of the overall inspection effort.

Supplementary Table 2: The 2015 estimated relative risks for each class of location. Scores provide a relative estimate of the likelihood of invasion into a single location of a given type. The values used come from the sum over all parasites (grey column).

Risk description	n	Relative risk: Foulbrood	Relative risk: Tropilaelaps	Relative risk: SHB	Relative risk: Σparasites	
Package/NUC importer	6	10	5	100	**115**	High Risk
High-volume queen importer (>100/year)	15	1	1	30	**32**	Medium Risk
Imported Honey Packer	25	15	1	3	**19**	
Crude hive products importer	12	3	1	15	**19**	
Fruit and vegetable wholesale market	25	1	1	15	**17**	
Plant importer	22	1	1	15	**17**	
Major Zoo	21	1	1	15	**17**	
Freight Port / Port	149	1	2	12	**15**	Low Risk
Freight depot	19	1	2	12	**15**	
Major international civilian airport	14	1	1	12	**14**	
Low volume queen importer (<100/year)	71	1	1	10	**12**	
Landfill from imported produce	3	10	1	1	**12**	
Military airport (American)	3	1	1	6	**8**	
Military airport (UK Forces)	23	1	1	4	**6**	
Civilian airport	33	1	1	2	**4**	
Quarantine Facility	1	1	1	2	**4**	
Area of influence (Radius in Km)		5 km	5 km	15 km	**5-15km**	

References

1 Haynes, E., Helgason, T., Young, J. P. W., Thwaites, R. & Budge, G. E. A typing scheme for the honeybee pathogen *Melissococcus plutonius* allows detection of disease transmission events and a study of the distribution of variants. *Environmental microbiology reports* **5**, 525-529 (2013).

2 Mill, A. C. *et al.* Clustering, persistence and control of a pollinator brood disease: epidemiology of American foulbrood. *Environmental Microbiology* **16**, 3753-3763, doi:10.1111/1462-2920.12292 (2014).

3 Beekman, M. & Ratnieks, F. Long-range foraging by the honey-bee, *Apis mellifera* L. *Functional Ecology* **14**, 490-496 (2000).

4 Anderson, H. & Marris, G. GB non-native organism risk assessment for *Tropilaelaps* spp.: *Tropilaelaps clareae* and *Tropilaelaps mercedesae*. (DEFRA, UK, 2011).

5 Evans, J. D., Pettis, J. S., Hood, W. M. & Shimanuki, H. Tracking an invasive honey bee pest: mitochondrial DNA variation in North American small hive beetles. *Apidologie* **34**, 103-109, doi:10.1051/apido:2003004 (2003).

6 Evans, J., Spiewok, S., Teixeira, E. & Neumann, P. Microsatellite loci for the small hive beetle, *Aethina tumida*, a nest parasite of honey bees. *Molecular ecology resources* **8**, 698-700 (2008).

7 Delaplane, K. S. Strictly for the hobbyist. *American Bee Journal* **138**, 884-886 (1998).

8 Dixon, D. & Lafreniere, R. The small hive beetle in Manitoba. *Manitoba Beekeeper*, 1 (2002).

9 Murilhas, M. A. *Aethina tumida* arrives in Portugal. Will it be eradicated? *EurBee Newsletter*, 3 (2005).

10 Somerville, D. Study of the small hive beetle in the USA (2003).

11 Anderson, H., Cuthbertson, A., Marris, G. & Wakefield, M. Pest Risk Analysis (PRA) for the small hive beetle, *Aethina tumida* (Murray) (Coleoptera: Nitidulidae). (DEFRA, UK, 2010).

12 Brown, M. *et al. Epidemiological surveillance and control of bee diseases*. (2014).

13 Wilkins, S., Brown, M. A. & Cuthbertson, A. G. S. The incidence of honey bee pests and diseases in England and Wales. *Pest Management Science* **63**, 1062-1068, doi:10.1002/ps.1461 (2007).
